# β-LacFamPred: An online tool for prediction and classification of β-lactamase class, subclass, and family

**DOI:** 10.3389/fmicb.2022.1039687

**Published:** 2023-01-12

**Authors:** Deeksha Pandey, Neelja Singhal, Manish Kumar

**Affiliations:** Department of Biophysics, University of Delhi South Campus, New Delhi, India

**Keywords:** antibiotic resistance, **β**-lactamase, *in silico* prediction tool, classification, hidden Markov models, high-throughput, annotation

## Abstract

β-Lactams are a broad class of antimicrobial agents with a high safety profile, making them the most widely used class in clinical, agricultural, and veterinary setups. The widespread use of β-lactams has induced the extensive spread of β-lactamase hydrolyzing enzymes known as β-lactamases (BLs). To neutralize the effect of β-lactamases, newer generations of β-lactams have been developed, which ultimately led to the evolution of a highly diverse family of BLs. Based on sequence homology, BLs are categorized into four classes: A–D in Ambler’s classification system. Further, each class is subdivided into families. Class B is first divided into subclasses B1–B3, and then each subclass is divided into families. The class to which a BL belongs gives a lot of insight into its hydrolytic profile. Traditional methods of determining the hydrolytic profile of BLs and their classification are time-consuming and require resources. Hence we developed a machine-learning-based *in silico* method, named as β-LacFamPred, for the prediction and annotation of Ambler’s class, subclass, and 96 families of BLs. During leave-one-out cross-validation, except one all β-LacFamPred model HMMs showed 100% accuracy. Benchmarking with other BL family prediction methods showed β-LacFamPred to be the most accurate. Out of 60 penicillin-binding proteins (PBPs) and 57 glyoxalase II proteins, β-LacFamPred correctly predicted 56 PBPs and none of the glyoxalase II sequences as non-BLs. Proteome-wide annotation of BLs by β-LacFamPred showed a very less number of false-positive predictions in comparison to the recently developed BL class prediction tool DeepBL. β-LacFamPred is available both as a web-server and standalone tool at http://proteininformatics.org/mkumar/blacfampred and GitHub repository https://github.com/mkubiophysics/B-LacFamPred respectively.

## Introduction

The rapid emergence of antimicrobial resistance (AMR) in bacteria due to the overuse of antibiotics is compromising the efficacy of antibiotics globally (Gould and Bal, 2013; Sengupta et al., 2013; Golkar et al., 2014; Wright, 2014). Unfortunately, the drying up of the new antibiotic development pipelines and the rapid spread of antibiotic resistance has become a significant global health crisis (Piddock, 2012; Bartlett et al., 2013; Gould and Bal, 2013; Gross, 2013; Sengupta et al., 2013; The antibiotic alarm, 2013; Lushniak, 2014; Michael et al., 2014; Read and Woods, 2014; Viswanathan, 2014). Bacteria employ multiple ways to neutralize the lethal effect of antibiotics. The most common mechanisms are (a) altering the permeability of cell membrane to stop/reduce the entry of antibiotics inside the cell, (b) enzymatic breakdown of antibiotics, (c) pumping out of drug molecules from the cell, and (d) altering the target of antibiotics.

β-lactams are the most commonly prescribed drug for treatment of Gram-negative bacterial infection. The resistance against β-lactam antibiotics is due to development of a highly diverse group of enzymes, collectively called β-lactamases (BLs), that hydrolyze the amide bond of a β-Lactam ring to make it ineffective (Abraham and Chain, 1940/1988; Petrosino et al., 1998; McKeegan et al., 2002; Zervosen et al., 2012). BLs is a highly diverged super-family of enzymes both in terms of sequence and functional diversity (Singh et al., 2009). Over the years, several classification systems have been developed to classify BLs. However, the most popular schemes are (i) Ambler’s classification scheme, which was based on the amino acid sequence similarity, and (ii) Bush, Jacoby, and Medeiros classification scheme, which was based on substrate and inhibitor profiles (Ambler, 1980; Bush et al., 1995; Mack et al., 2020). Ambler’s classification scheme categorized BLs into four classes: A–D. Class A, C, and D are also known as serine BLs because they have an active-site serine to catalyze the hydrolysis. Class B BLs is known as Metallo β-lactamases (MBLs) since they use zinc ions (Zn2+) for their activity (Galleni et al., 2001). MBLs are distinct from the serine BL in sequence, structure fold, and catalytic mechanism (Bush, 1998) and they are further divided into three subclasses, B1–B3, based on their active site geometry and overall homology (Walsh et al., 2005).

The Bush, Jacoby, and Medeiros classification scheme attempted to correlate the phenotype of clinical isolates with substrate and inhibitor profiles. It classified BLs into three major groups: Group 1 BLs (class C BLs) is cephalosporinases that are not well inhibited by clavulanic acid; Group 2 (classes A and D) is the largest group of BLs. It includes penicillinases, cephalosporinases, and broad spectrum BLs generally inhibited by active site-directed BL inhibitors; Group 3 are MBLs that hydrolyze penicillins, cephalosporins, and carbapenems but are poorly inhibited by a majority of beta-lactam containing molecules (Petrosino et al., 1998; Zervosen et al., 2012). Based on the differences among the enzymes, each group is further divided into several subgroups and families (Petrosino et al., 1998). Diversity in the amino acid sequences of different BL families also affects the clinical outcome. The family of BL ultimately decides whether the prescribed β-lactam antibiotics would be able to kill the drug-resistant pathogen infection or not.

Several screening tests have been developed to identify the family of BLs at both gene and whole genome levels (Livermore et al., 2001; Sharma et al., 2004). However, these methods are resource and time-consuming. An alternative approach for rapid annotation of BLs family is to use computational methods (Moradigaravand et al., 2018), which can quickly identify BLs genes/proteins and classify them into the family. The most popular computational approach is using BLAST search against either general-purpose molecular biology databases such as NCBI NR/NT or UniProtKB/SwissProt or BL-specific databases such as BLDB (Naas et al., 2017), BLAD (Danishuddin et al., 2013), LacED (Thai et al., 2009), ARDB (Liu and Pop, 2009), CARD (McArthur et al., 2013; Jia et al., 2017), and CBMAR (Srivastava et al., 2014b). Other approaches to predict, classify, and annotate BLs and/or its families are by prediction of family-specific motifs or patterns using LactFP (Srivastava et al., 2014a) or by using machine learning-based algorithms such as Bayes (Nath and Karthikeyan, 2017), support vector machine (SVM) (Kumar et al., 2015; Srivastava et al., 2018), convolutional neural network (CNN) (White et al., 2017) VGGNet architecture and TensorFlow deep learning (Wang et al., 2021). Machine learning-based algorithms present an opportunity for increasing the sensitivity of classification over alignment and thus have been previously used with high-throughput sequence data to characterize the resistome. For example, AMRFinderPlus (Feldgarden et al., 2021) and Meta-MARC (Lakin et al., 2019) used Hidden Markov Models (HMMs) to classify AMR-related protein/gene sequences from high-throughput sequence data and sequence reads. The existing methods and databases, both specific for BLs and general purpose, have made significant contributions in annotation of different variants of BLs. However, most prediction methods except LactFP were restricted only to the prediction up to class level [e.g., βLact-Pred (Ashraf et al., 2021), CNN-BLPred (White et al., 2017), PredLactamase (Kumar et al., 2015)], or subclass [e.g., BlaPred (Srivastava et al., 2018)]. LactFP predicts the class, sub-class, and family of a BL protein on the basis of presence of a family-specific motif called fingerprint in the primary amino acid sequence. However, there are a few limitations of LactFP. The most critical limitation of LactFP was that it was developed using a dataset compiled in 2014. Over time information about new family members and many mutations in different families has been accumulated in the databases. Hence LactFP might not be capable of predicting all BL families correctly. This indicates that a tool capable of predicting more BL families is the need of the hour.

**Figure 1:**
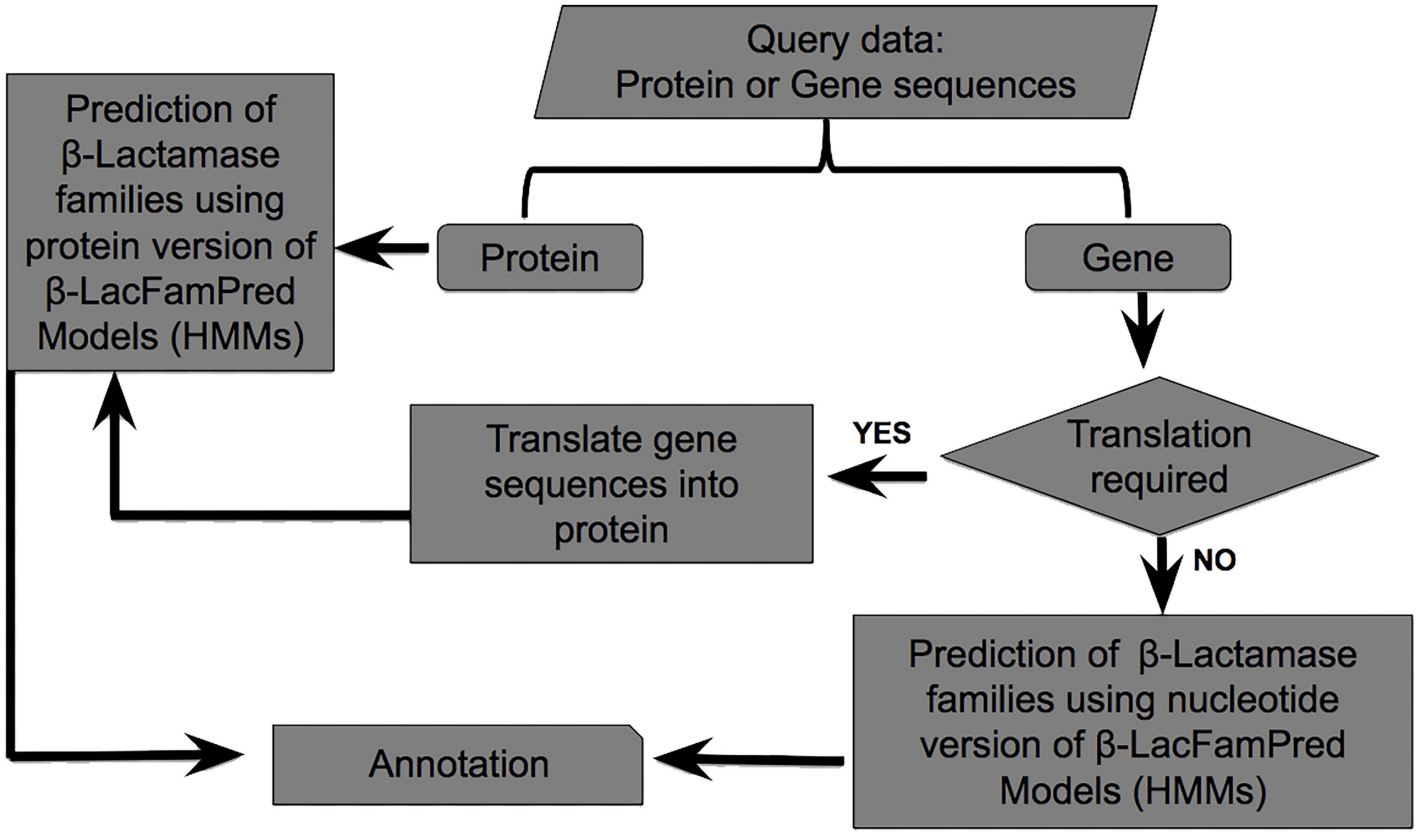
Prediction schema of β-LacFamPred tool.

To address the above-mentioned limitations, we present β-LacFamPred, a machine learning based classifier that can annotate BLs up to the family level. β-LacFamPred can be used on both genomic and proteomic data. To develop β-LacFamPred, the data were initially extracted from two BL databases namely, CBMAR and BLDB. Afterward, new sequences were added to each family from UniProtKB and NCBI NR. Using these sequences we constructed 96 HMMs, each specific for one family of BL. The consistency of prediction of each HMM was evaluated using leave-one-out approach. We benchmarked the effectiveness of β-LacFamPred on an independent dataset vis-a-vis LactFP and observed very high performance (≥98% precision & recall and ≥ 99% accuracy). We also developed a user-friendly web-server of β-LacFamPred that is available at http://proteininformatics.org/mkumar/blacfampred. The working schema of β-LacFamPred is shown in Figure 1.

## Materials and methods

### Training and benchmarking independent dataset

The family-wise sequences of BLs were obtained from our earlier developed database, Comprehensive β-Lactamase Molecular Annotation Resource (CBMAR) and BLDB. The number of protein sequences in each family was also augmented from CARD, UniProtKB, and NCBI NR databases. Sequences of each family were manually curated using literature and UniProtKB annotations. We also removed the fragmented sequences from each family. BL families with less than five sequences or single sequences were also removed from further studies. If multiple copies of identical sequences were present in a family, then all except one sequence were removed. Finally, we found 96 BL families consisting of full-length sequences only. The number of sequences at each stage of data compilation is shown in Table 1.

**Table 1:** Statistics of BL families retrieved from CBMAR and BLDB databases.

Class	Sub-class	Total families	Families with one sequence	Families with <5 sequences	Families with ≥5 sequences
A	–	64# + 13* = 77	17#	0	47# + 13* = 60
B	B1	20# + 35* = 55	11# + 31* = 42	3*	9# + 1* = 10
	B2	3# + 3* = 6	2# + 2* = 4	1*	1#
	B3	13# + 42* = 55	9# + 38* = 47	1# + 3* = 4	3# + 1* = 4
C	–	14# + 9* = 23	4#	0	10# + 9* = 19
D	–	2# + 18* =20	18*	0	2#
Total		236	132	8	96

#: CBMAR, *: BLDB.

We used the reference *bla* gene sequences obtained from Lee et al. (2015) for benchmarking. These BL sequences were used to develop molecular probes for PCR-based methods to detect *bla* genes in various pathogenic isolates (Lee et al., 2015). The total number of *bla* gene sequences were 1,342, belonging to all four Ambler’s classes, A–D, and 29 families of BLs.

### Construction of the β-LacFamPred HMMs

Sequences of each BL family were multiply aligned using the Muscle 3.8 program (Edgar, 2004) at default parameters. Using the hmmbuild function of the HMMER tool (version 3.1) (Finn et al., 2011), we build HMM of each BL family.

### Cross-validation and performance metrics

To test the efficiency of each HMM in discriminating between the family and non-family members, we used the leave-one-out cross-validation (LOOCV) approach. During LOOCV, HMM was built using all but one family sequence for each BL family. Hence the total number of HMMs built for a family during LOOCV was equal to the number of BL sequences in that family. The performance of each model was evaluated against a dataset containing (a) excluded sequence of a particular family for which HMM was being evaluated and (b) sequences using which remaining 95 HMMs were built. The search efficiency of each HMM was determined based on the best hit, i.e., search results having a minimum e-value. If the best search result was the left-out sequence of the same family, the search result was categorized as True Positive (TP). If the best search result belonged to a different BL family, the search result was categorized as False Positive (FP). The performance of methods was assessed using the standard evaluation metrics namely, precision, recall, accuracy, and *F*-measure. These performance metrics have also been frequently used in several prediction and classification studies (Pandey et al., 2020). For example, during prediction for a BL protein that belongs to a hypothetical β-lactamase family ‘X’, if it is predicted to belong to the same class ‘X’, the prediction was categorized as TP prediction; if it were predicted to class non-‘X’, it would be an FN prediction.

Similarly, if a non-‘X’ is predicted as non-‘X’ and ‘X’, it is an example of TN and FP predictions, respectively. TP, TN, FP, and FN represent true positives, true negatives, false positives, and false negatives, respectively. The expressions used to calculate the above-mentioned parameters were:


(1) $$\mathrm{Precision}=\frac{\mathrm{TP}}{\mathrm{TP}+\mathrm{FP}}\times 100$$

(2) $$\;\mathrm{Recall}=\frac{\mathrm{TP}}{\mathrm{TP}+\mathrm{FN}}\times 100$$

(3) $$F\mathrm{-measure}=2\times \left(\frac{\mathrm{Precision}\times \mathrm{Recall}}{\mathrm{Precision}+\mathrm{Recall}}\right)$$

(4) $$\mathrm{Accuracy}=\frac{\mathrm{TP}+\mathrm{TN}}{\mathrm{TP}+\mathrm{FP}+\mathrm{TN}+\mathrm{FN}}\times 100$$

The precision of each HMM was determined as the ratio of the number of proteins whose family was correctly predicted to the total number of proteins, which were predicted as a member of that BL family (Eq. 1). A precision value of 1.0 of an HMM indicates the correct prediction of all family proteins. Recall of an HMM was the ratio of proteins whose family was correctly predicted to the number of proteins in that BL family (Eq. 2). The recall value of 1.0 indicates that all proteins of that family were correctly predicted. The overall percentage of correctly predicted examples is calculated through accuracy (Eq. 3). To balance the precision and recall values due to unequal composition of family (positive) and non-family (negative) sequences during evaluation, *F*-measure was used, which is the harmonic mean of precision and recall (Powers, 2020).

### Functional annotation

All 96 BL HMMs were annotated using (a) ARG databases, namely DeepARG – ARGminer (Arango-Argoty et al., 2018), CARD, ARDB, (b) UniProtKB, and (c) published research papers. The annotation details mentioned with each HMM are resistance mechanisms, class, and name of antibiotic against which the family confers the resistance, family, class, subclass, and phenotypic information as per Jacoby and Bush classification scheme. Each HMM was also tagged with the information of their action in terms of their spectrum, namely broad spectrum, extended spectrum, and narrow spectrum (Supplementary Table S1). β-LacFamPred webserver is freely available at http://proteininformatics.org/mkumar/blacfampred.

### Comparative evaluation

We compared the performance of β-LacFamPred with well-known ARG annotation methods: AMRFinderPlus, RGI-CARD, ResFinder, and Meta-MARC. We have also included LactFP as it assigns the family of a BL sequence based on the presence of a conserved motif. The comparative evaluation was done on the independent test dataset consisting of 1,342 BL protein/gene sequences that belong to 29 BL families.

## Results and discussion

### Description of data and β-LacFamPred models

The class-wise distribution of BL families in CBMAR was as follows: Class A = 64, Class B = 36, Class C = 14, and Class D = 2. In BLDB, the number of Class A–D families, which were not present in CBMAR, was 13, 80, 9, and 18, respectively (Table 1). The total number of BL families with which we started the work was as follows: Class A = 77, Class B = 116, Class C = 23, and Class D = 20. After removing families that had less than five sequences we were left with 96 families, of which 60, 15, 19, and 2 belonged to classes A–D, respectively. The subfamily-wise distribution of class B families were 10, 1 and 4 in subclass B1–B3, respectively. In the final dataset, the total number of BL families obtained from CBMAR and BLDB was 72 and 24, respectively, with at least five sequences. We did pairwise multiple sequence alignment of sequences of each BL family and converted the alignment HMM. The current version contained 96 BL HMMs.

### Cross-validation of β-LacFamPred models

The consistency in search efficiency of all 96 HMMs was evaluated using the leave-one-out cross-validation (LOOCV) approach. We calculated the true positive and false positive prediction rate of each BL HMM of β-LacFamPred models. A detailed description of the LOOCV method was described in the materials and methods section under the sub-heading cross-validation and performance metrics. During LOOCV, all 96 HMMs gave 100% accuracy. In summary, the cross-validation (LOOCV) showed that all 96 HMMs could predict the family of BL with very high consistency and efficiency. The complete set of 96 HMMs capable of predicting 96 families of BLs is called β-LacFamPred henceforth.

### Performance evaluation on independent datasets

In class A BL, except for one protein of family TEM, all were correctly predicted to their actual family and achieved 100% accuracy and 100% precision, recall, and *F*-measure in all cases of class A (Table 2). The wrongly predicted class A *bla* gene originally belonged to TEM, but our method wrongly predicted it as a CTXM family member. In the case of TEM, we have found accuracy was 99 and 99.38% precision, recall, and F1 score. During performance evaluation on the independent dataset we got the hits of CTX-M family against KLUA, KLUY, KLUC, TOHO and CTX-M. To find the reason behind this we did a profile HMM-HMM comparison using (hhalign module of HMMER) using the KLUA, KLUY, KLUC, TOHO and CTX-M, which showed they are highly similar to each other. To further check the evolutionary relationship among them, we also constructed a phylogenetic tree using profile HMMs that showed that CTX-M and KLUC shared the same branch but KLUA and TOHO did not belong to the same branch. We also saw KLUY forming a separate branch, which was shown in Supplementary Figure S1. Due to this we added these sequences to a separate family. In addition to this, we have also noticed that several databases mention these families as separate. We have marked the ‘CTX-M like families’ in front of KLUY, KLUA, KLUC and TOHO families.

**Table 2:** Performance evaluation of **β**-LacFamPred on an independent dataset.

Ambler’s class	Actual gene type	Precision (%)	Recall (%)	*F*-measure (%)	Accuracy
A	GES/IBC	100%	100%	100%	1
	TEM	99.38%	99.38%	99.38%	0.99
	NMC/IMI	100%	100%	100%	1
	KPC	100%	100%	100%	1
	SHV/LEN/OKP	100%	100%	100%	1
	PER	100%	100%	100%	1
	SME	100%	100%	100%	1
	CTXM/KLUA/KLUY/TOHO	100%	100%	100%	1
B	SIM	100%	100%	100%	1
	IND	100%	100%	100%	1
	IMP	100%	100%	100%	1
	NDM	100%	100%	100%	1
	VIM	100%	100%	100%	1
	BlaB	100%	100%	100%	1
	DIM	100%	100%	100%	1
	TMB	100%	100%	100%	1
	GIM	100%	100%	100%	1
	CphA	100%	100%	100%	1
	GOB	100%	100%	100%	1
C	ACC	100%	100%	100%	1
	DHA	100%	100%	100%	1
	ACT/MIR	100%	100%	100%	1
	PDC	100%	100%	100%	1
	ADC	100%	100%	100%	1
	LAT	100%	100%	100%	1
	SRT/SST	100%	100%	100%	1
	CMY/MOX/FOX/AmpC	92.30%	92.30%	92.30%	0.99
	EC	100%	100%	100%	1
D	OXA	99.52%	99.52%	99.52%	0.99

In the case of class B BLs, all sequences were correctly predicted to their respective family; hence the number of false predictions was zero, and accuracy was also found to be 100%. In the case of class C BLs in eight of nine families, no false prediction was observed. The accuracy found in all eight cases was 100%. However, in one family, namely CMY/MOX/FOX/AmpC, there were 39 sequences, out of which 36 were correctly predicted as members of CMY/MOX/FOX/AmpC.

In contrast, three sequences were wrongly predicted to belong to family AQU, and the accuracy we achieved, in that case, was 99% and 92.30 precision, recall, and F1 score. In the case of class D BLs, out of 210 sequences, 209 BL sequences were correctly predicted as OXA family members. At the same time, one was incorrectly predicted as a member of the IMP family, and in that case, also we found 99% accuracy and 99.52 precision, recall, and F1 score, respectively (Table 2).

To further assess the capability of β-LacFamPred for identifying BL class, subclass, and families, we performed an additional independent evaluation using a Penicillin-Binding Proteins (PBPs) dataset. PBPs are membrane-associated proteins involved in the biosynthesis of peptidoglycan components of bacterial cell walls. PBP and BLs belong to the superfamily of serine penicillin-recognizing enzymes and have similar conserved protein folds (Knox et al., 1996; Meroueh et al., 2003). PBP and BLs are homologous proteins, but PBP does not provide antibiotic resistance against BLs. Also, BLs is considered to have evolved from penicillin-binding proteins. PBPs were not part of the dataset on which β-LacFamPred prediction models were developed. Out of 60 PBP sequences, only four were wrongly predicted as BLs. Three sequences were predicted as a member of the family AmpC (class C) and one sequence to family ARL (class A).

To further confirm the discriminatory capability of β-lactamase, and non-β-lactamase, we created a second independent dataset consisting of glyoxalase II, which belongs to the metallo-beta-lactamase (MBL) superfamily of proteins. The sequences of the glyoxalase II were retrieved from the UniProtKB database. We found a total of 57 full-length sequences of glyoxalase II. At e-value 1e-15 none of the glyoxalase II sequences were predicted as BL. When e-value was increased to 1e-10, 1e-6 and 0.1 the number gradually increased to 17, 43 and 43, respectively. The result was consistent with previous work that had shown the requirement of more stringent e-value cutoff to reduce the number of false positive predictions (Zankari et al., 2012; McArthur et al., 2013; Gibson et al., 2015).

### Proteome-wide screening of β-lactamases

Recently Wang et al. have developed a deep learning based method, DeepBL, for predicting and classifying BLs on the basis of their protein sequences (Wang et al., 2021). To characterize the complete repertoire of BLs, they annotated all reviewed bacterial protein sequences (334,542 in total) from the UniProtKB database. DeepBL identified 2,876 Class-A, 665 Class-B, 335 Class-C, and 231 Class-D BL protein sequences in this dataset. To examine the capability of β-LacFamPred in predicting BLs in screening of a proteome-wide high-throughput data, we also annotated the 334,542 protein sequences, compiled by DeepBL, using β-LacFamPred. We found 86 Class-A, 246 Class-B, 67 Class-C, and 29 Class-D BL proteins. Further, 86 Class-A BLs were classified into 26 families. Out of 246 Class-B proteins, 21 were predicted as subclass B1, 02 were predicted as subclass B2, and 223 were predicted to belong to subclass B3, which were further classified into different families as follows: B1 = 5, B2 = 1 and B3 = 3. In the case of 67 and 29 proteins, which were predicted to belong to class C and D respectively, they were predicted to belong to 10 and 2 families, respectively (Table 3; Supplementary Table S2). Since there was a significant difference in the number of proteins predicted as BLs by DeepBL and β-LacFamPred, we compared the UniProtKB annotations with predictions of BLs by DeepBL. We observed that out of 4,107, and 428 proteins predicted as BL by DeepBL and β-LacFamPred, only 199 and 252 were annotated as BLs by the UniProtKB database (Table 3; Supplementary Table S3).

**Table 3:** Number of proteins predicted as BL by DeepBL and **β**-LacFamPred and annotation statistics of UniProtKB therein.

Ambler’s class	Number of proteins predicted as BL by DeepBL/annotated as BL by UniProtKB	Number of proteins predicted as BL by β-LacFamPred/annotated as BL by UniProtKB	Number of class B predicted as BL and their sub-class prediction	Number of families in which predicted BLs were distributed as per β-LacFamPred
A	2876/80	86/77	–	26
B	665/91	246/145	21 (B1)	5
			2 (B2)	1
			223 (B3)	3
C	335/13	67/15	–	10
D	231/15	29/15	–	2
Total	4107/199	428/252	246	47

When we analyzed the prediction results of DeepBL and β-LacFamPred about the UniProtKB annotations, we found the four situations. A few examples are shown in Table 4 as an illustration. Four different situations we found were (a) both DeepBL and β-LacFamPred correctly predicted the nature and class of query protein (Sr. No 1–10 of Table 4), (b) DeepBL predicted non-BL but β-LacFamPred predicted the query sequence as BL and also predicted its corresponding family. The UniProtKB annotation also supported β-LacFamPred annotations (Sr. No 11–12 of Table 4), (c) DeepBL predicted the query sequence as BL, but β-LacFamPred predicted them as non-BL. UniProtKB also supported the β-LacFamPred prediction (Sr. No 13 of Table 4), and (d) both DeepBL and β-LacFamPred predicted the query sequence as non-BL and UniProtKB also supported the prediction (Sr. No 14–15 of Table 4). The results showed that the number of false positive predictions in β-LacFamPred was significantly lower than DeepBL and β-LacFamPred can be used to predict and annotate new BLs that are not known yet.

**Table 4:** Comparative prediction outputs of DeepBL, UniProtKB and **β**-LacFamPred.

S. No.	ID	Prediction tools					
		DeepBL	UniProtKB		β-LacFamPred		
1.	Q9EZQ7	Class A	Class-A beta lactamase	Beta-lactamase AST-1	Class A	–	AST
2.	Q9S424	Class A	Class-A beta-lactamase	Beta-lactamase SHV-13	Class A	–	SHV
3.	P28585	Class A	Class-A beta-lactamase	Beta-lactamase CTX-M-1	Class A	–	CTXM
4.	O08498	Class B	Class-B beta-lactamase	Metallo-beta-lactamase BlaB1	Class B	Sub-class B1	BLAB
5.	C7C422	Class B	Class-B beta-lactamase	Metallo-beta-lactamase blaNDM-1	Class B	Sub-class B1	NDM
6.	P26918	Class B	Class-B beta-lactamase	Metallo-beta-lactamase Type 2 cphA	Class B	Sub-class B2	CPHA
7.	A0A096ZEC9	Class A	Class-B beta-lactamase	Metallo-beta-lactamase Type 2 cphA	Class B	Sub-class B2	CPHA
8.	O05465	Class C	Class-C beta-lactamase	Beta-lactamase ampc	Class C	–	AmpC
9.	B3U538	Class D	Class-D beta-lactamase	Beta-lactamase OXA-133	Class D	–	OXA
10.	Q00983	Class D	Class-D beta-lactamase	Beta-lactamase LCR-1	Class D	–	LCR
11.	A6V707	Not beta-lactamase	Class-B beta-lactamase	Metallo-beta-lactamase	Class B	Sub-class B3	L1
12.	O31760	Not beta-lactamase	Class-B beta lactamase	Metallo-beta-lactamase	Class B	Sub-class B1	IMP
13.	A0A0H2UR93	Class A	Glucosyltransferase 3	Gtf3 glucosyltransferase family	Non Beta-lactamase		
14.	B6I4P3	Not beta-lactamase	L-rhamnose mutarotase	Rhamnose mutarotase family	Non Beta-lactamase		
15.	V6F4W4	Not beta-lactamase	Magnetosome protein MamZ	Major facilitator superfamily	Non beta-lactamase		

Table 4 also showed the advantage of β-LacFamPred over DeepBL. Whereas DeepBL can predict only up to Ambler’s class of query BL, β-LacFamPred can predict both the class and family of query BL and subclass in the case of class B BL.

### Performance comparison with existing methods

Out of 1,342 BL sequences of independent dataset AMRFinderPlus, RGI-CARD, ResFinder, Meta-MARC, LactFP, and β-LacFamPred correctly predicted 1,026, 1,115, 1,242, 1,199, 742, and 1,320 BL sequences to their respective families Table 5. Family-wise performance of each method is shown in Supplementary Tables S4–S10. AMRFinderPlus obtained accuracy ranges from 1 to 0.95 in most BL families. Only in a few families, namely, SHV/LEN/OKP, GIM, and EC no correct prediction was done. In RGI-CARD, we obtained accuracy ranges from 1 to 0.92 in different BL families, except in the case of GIM and LAT, we found zero accuracy. Among all methods, LactFP showed the least performance. In 25 BL families, the accuracy range was 100 to 79%. In families GOB, PDC, ADC, and EC all proteins were correctly predicted (Supplementary Tables S7–S10). In Meta-MARC, accuracy ranges from 1 to 0.98 in different BL families, but in several cases, zero accuracy was also observed (SIM, DIM, TMB, GIM, and EC) (Supplementary Table S4). ResFinder predicted 28 families in a range of 98–100% accurately except for one family (EC) of class C, where it predicted the wrong family of all 87 sequences. Compared to other methods, our proposed method achieves a very high accuracy (ranges from 1 to 0.99) in all 29 families on an independent dataset of 1,342 BL gene datasets compared to other existing methods in Supplementary Table S6.

**Table 5:** Comparison of proposed method **β**-LacFamPred with existing methods.

Method	Type of data	TP	FP	TN	FN	Precision (%)	Recall (%)	*F*-measure (%)	Accuracy
β-LacFamPred	Protein sequences	1,320	22	37,554	22	98.36%	98.36%	98.36%	0.99
RGI-CARD		1,115	227	37,349	227	83.08%	83.08%	83.08%	0.98
AMRFinderPlus		1,026	316	37,260	316	76.45%	76.45%	76.45%	0.99
LactFP		742	600	36,976	600	55.29%	55.29%	55.29%	0.96
β-LacFamPred	Gene sequences	1,337	5	37,571	5	99.62%	99.62%	99.62%	0.99
Meta-MARC		1,199	143	37,433	143	89.34%	89.34%	89.34%	0.99
ResFinder		1,242	100	37,476	100	92.54%	92.54%	92.54%	0.99

Moreover, we were unable to predict the BLs in the gene/protein independent dataset using the web servers of AMRFinderPlus, Meta-MARC, RGI-CARD, ResFinder, and LactFP, due to the limitations of their web services (The first two methods not available in the form of web servers which raises difficulty in handling for non-programmers, the third one allows a maximum of 20 Mb limit size of sequences per submission for prediction. ResFinder only allows genomic sequences as an input for predicting ARGs, not a proteome, and LactFP has a web server. However, it only deals with a single protein sequence for prediction, while β-LacFamPred provides a user-friendly web server and standalone tool for protein and gene sequence analysis. The web server of β-LacFamPred allows users to analyze 100 gene/protein sequences in one go). For proteome/genome/metagenome scanning. We also provided a standalone version of our tool for predicting families of BLs. The comparison clearly demonstrated better capability of BLs class, subclass, and family prediction of β-LacFamPred in comparison to other ARG annotation tools.

### Advantages and limitations of present and previously developed BL family prediction method

In the past, we developed a motif-based prediction method of BL classification named LactFP. Although LactFP and β-LacFamPred are based on BL family datasets, they were developed using different approaches. There are also several advantages of β-LacFamPred over LactFP. For example, (a) LactFP was developed using 71 families, 46 from class A, 15 from class B (10 from subclass B1, one from subclass B2, four from subclass B3), eight from class C, and two from class D. On the other hand, β-LacFamPred was developed using 96 families, 60 from class A, 15 from class B (10 from subclass B1, one from subclass B2, four from subclass B3), 19 from class C, two from class D. (b) In LactFP fingerprints were extracted by using only 605 protein sequences. In contrast, β-LacFamPred models were built using >8,000 protein sequences. (c) The total 605 protein sequence dataset of LactFP contained 325 sequences in class A, 58 sequences in subclass B1, 14 sequences in subclass B2, 58 sequences in subclass B3, 139 sequences in class C, and 11 sequences in class D, while the complete 8,060 protein sequences of β-LacFamPred contained 4,404 from class A, 682 from subclass B1, 67 from subclass B2, 132 from subclass B3, 2,438 from class C, 337 from class D. (d) The LactFP web server allows users to search the family-specific fingerprint in only protein sequences while the β-LacFamPred web server is capable of handling both protein/gene sequences or complete proteome/genome. Additionally, β-LacFamPred provides annotation along with the family information, which is not available in LactFP. (e) The family-specific patterns/motifs-based tool LactFP only identifies short conserved sequence patterns. In contrast, the domain-based HMM tool β-LacFamPred identifies more extended conserved regions in a protein or gene (Table 6).

**Table 6:** Advantages and limitations of LactFP and **β**-LacFamPred.

Feature	LactFP	β-LacFamPred
Training data source	UniProtKB/TrEMBL	CBMAR, BLDB, CARD, UniProtKB, NCBI NR/NT
Total dataset	605	8,060
Less than 5 sequence family used	Yes	No
One sequence family used	No	No
Similarity tool and threshold used	Blast (1e-4)	Blast (1e-6)
Total families	71	96
Benchmark data source	None	Lee et al. (2015)
Data redundancy threshold	Not mentioned	CD HIT (100%)
Tool used to develop prediction model	Meme/Mast	HMM
Cross-validation method	No	Leave-one-out cross validation (LOOCV)
Web server	Yes	Yes
Input data	Only Protein sequences	Protein/Gene sequences
Standalone availability	No	Yes
Advanced search options	No	Yes
Annotation provides against query	No	Yes

### Description of the β-LacFamPred web-server and standalone tool

**Figure 2:**
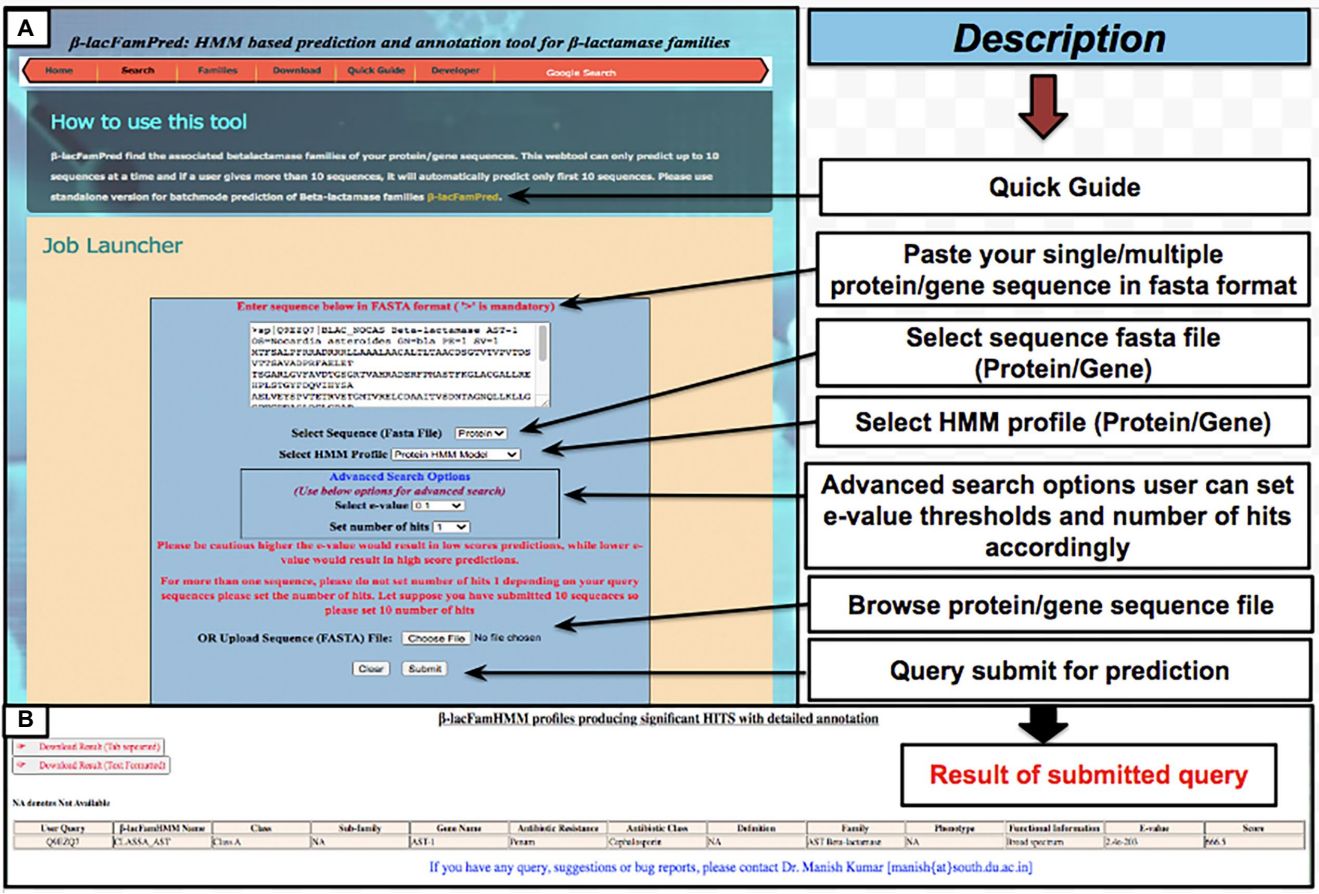
Screenshots of web pages of ‘β-LacFamPred’ tool **(A)** search page **(B)** prediction result page.

**Figure 3:**
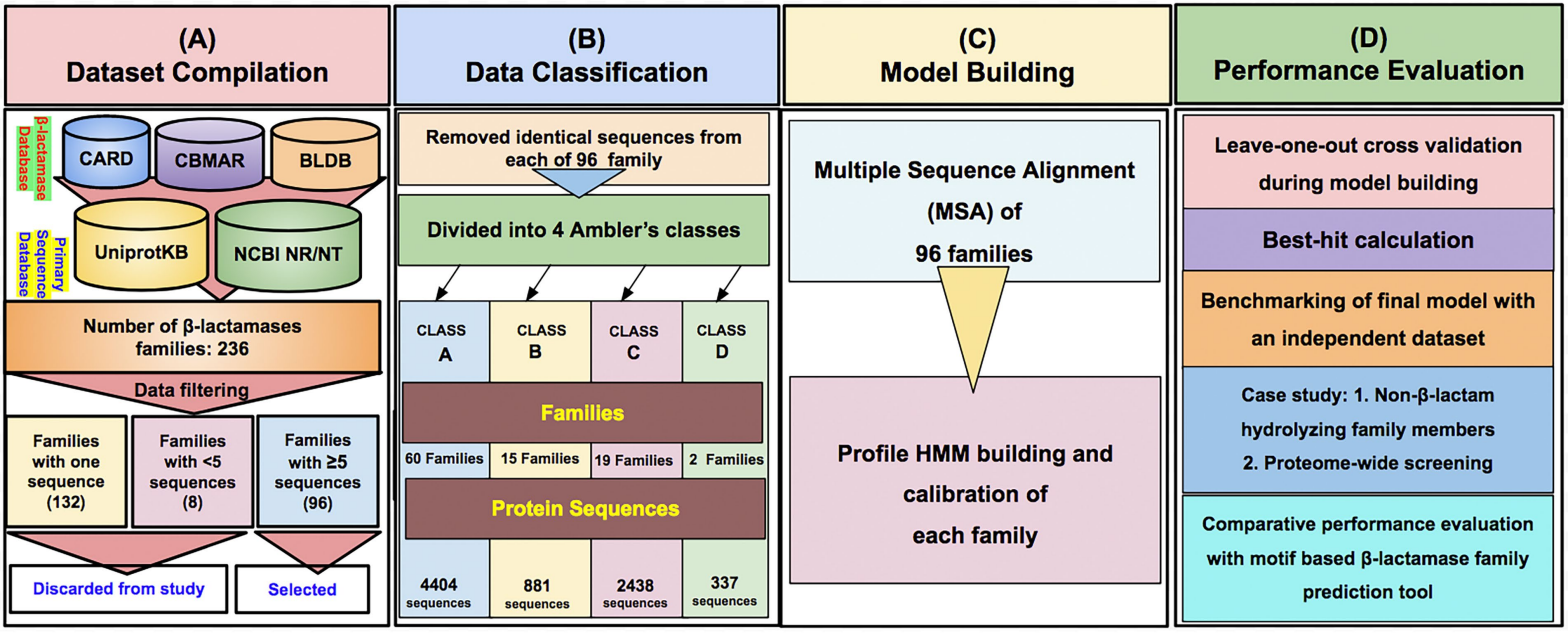
The overall workflow depicting the methodology used for designing the profile HMMs-based prediction tool β-LacFamPred. **(A–D)** represents the series of workflow.

To provide the β-LacFamPred prediction module to the scientific community, we also established a web server that can be used to query whether a gene/protein sequence is BL or not. If the query sequence is predicted as BL, then its probable class/subclass and families, along with other annotation details, will also be provided. The overall schema of the prediction methodology of the tool is explained in Figure 1. The β-LacFamPred web server can process a maximum of 100 sequences in one go. A snapshot of the search and prediction page of the ‘β-LacFamPred’ webserver is shown in Figure 2. The overall workflow depicting the methodology adopted for designing the β-LacFamPred tool has been illustrated in Figure 3. For high-throughput, whole genome, metagenomic and proteome/genome-scale annotation, a standalone version would be required, which is provided in the download section of the webserver at: http://proteininformatics.org/mkumar/blacfampred/download.html.

### Utility of β-LacFamPred

Recent advancements in sequencing technologies have produced a large amount of data that might contain novel variants of BLs. As experimental characterization and annotation is an expensive and time-consuming exercise hence to facilitate rapid annotation of potential BLs, we developed an *in silico* tool named β-LacFamPred. It can predict BL and their families using only protein/gene sequences. β-LacFamPred can also be combined with traditional surveillance methods and thus can complement the traditional BL families’ annotation methods. The current version of β-LacFamPred can predict 96 BL families. In the near future, we strive to update the β-LacFamPred tool regularly to reflect the latest discoveries of BL families. We hope that β-LacFamPred would help in annotation of the novel BLs genes/proteins and help in the progress of studies related to BL-based antimicrobial resistance.

## Conclusion

We reported an *in silico* tool, β-LacFamPred, for annotation and prediction of BLs in classes, subclasses, and families. Evaluation and comparison with other methods using independent datasets and proteome-wide screening showed β-LacFamPred to be a highly efficient tool.

## Data availability statement

The original contributions presented in the study are included in the article/Supplementary material, further inquiries can be directed to the corresponding author.

## Author contributions

DP collected and organized the data and developed the web interface. DP and MK analyzed the results. DP, NS, and MK wrote the manuscript. MK conceived the idea and did overall supervision of the work. All authors contributed to the article and approved the submitted version.

## Funding

DP was supported by the Department of Science and Technology (INSPIRE Program) (DST INSPIRE Fellowship/2016/IF160262) [Grant Number: DST/INSPIRE 03/2015/003022]. NS was supported by the Council of Scientific and Industrial Research under the Pool Scientist Scheme [Grant Number: 13(9089-A)/2019-POOL]. The work was carried out using the resources funded by the Indian Council of Medical Research [Grant Numbers: VIR (25)/2019/ECD-1 and ISRM/12(33)/2019].

## Conflict of interest

The authors declare that the research was conducted in the absence of any commercial or financial relationships that could be construed as a potential conflict of interest.

## Publisher’s note

All claims expressed in this article are solely those of the authors and do not necessarily represent those of their affiliated organizations, or those of the publisher, the editors and the reviewers. Any product that may be evaluated in this article, or claim that may be made by its manufacturer, is not guaranteed or endorsed by the publisher.

## Supplementary material

Supplementary Figure S1 Phylogenetic tree of Class A beta-lactamase families.
