# Supplementary figures and images for: β-LacFamPred: An online tool for prediction and classification of β-lactamase class, subclass, and family
Source: Front Microbiol. 2023 Jan 12;13:1039687. doi: 10.3389/fmicb.2022.1039687 (PMC9878453; doi:10.3389/fmicb.2022.1039687)

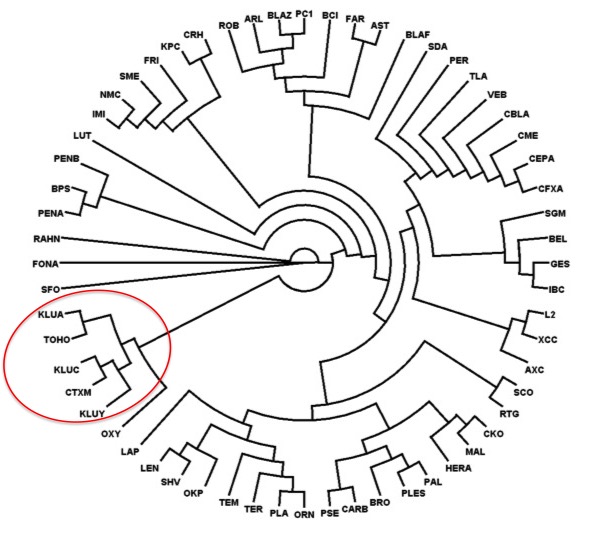

Supplement: Supplementary Figure S1 — Phylogenetic tree of Class A beta-lactamase families. [file Image_1.jpeg]
